# Two-Dimensional Ordering of Solute Nanoclusters at a Close-Packed Stacking Fault: Modeling and Experimental Analysis

**DOI:** 10.1038/srep07318

**Published:** 2014-12-04

**Authors:** Hajime Kimizuka, Shu Kurokawa, Akihiro Yamaguchi, Akira Sakai, Shigenobu Ogata

**Affiliations:** 1 Department of Mechanical Science and Bioengineering, Osaka University, Osaka 560-8531, Japan; 2 Department of Materials Science and Engineering, Kyoto University, Kyoto 606-8501, Japan; 3 Center for Elements Strategy Initiative for Structural Materials, Kyoto University, Kyoto 606-8501, Japan

## Abstract

Predicting the equilibrium ordered structures at internal interfaces, especially in the case of nanometer-scale chemical heterogeneities, is an ongoing challenge in materials science. In this study, we established an *ab-initio* coarse-grained modeling technique for describing the phase-like behavior of a close-packed stacking-fault-type interface containing solute nanoclusters, which undergo a two-dimensional disorder-order transition, depending on the temperature and composition. Notably, this approach can predict the two-dimensional medium-range ordering in the nanocluster arrays realized in Mg-based alloys, in a manner consistent with scanning tunneling microscopy-based measurements. We predicted that the repulsively interacting solute-cluster system undergoes a continuous evolution into a highly ordered densely packed morphology while maintaining a high degree of six-fold orientational order, which is attributable mainly to an entropic effect. The uncovered interaction-dependent ordering properties may be useful for the design of nanostructured materials utilizing the self-organization of two-dimensional nanocluster arrays in the close-packed interfaces.

Structural transformations at interfaces are of fundamental interest as complex examples of phase transitions in low-dimensional systems (see, for example, Refs. 1,2,3). Recent works have shown that a variety of interfaces such as grain boundaries, heterophase boundaries, and surfaces can be considered as separate, quasi-two-dimensional “phases” that may undergo phase-like transitions in which their structural and chemical properties change abruptly at critical values of the thermodynamic parameters^4^,^5^,^6^,^7^. Such interfacial phases (termed “complexions”) are not intrinsically stable as stand-alone phases but are thermodynamically stabilized by the boundaries provided by the adjoining bulk regions. If a significant fraction of the interfaces in a material undergoes a transition, the cumulative effect can cause a dramatic change in the macroscopic properties of the material. However, the nature of the interfaces is highly complex, and it has been an ongoing challenge to link material performance to the internal interface structure and the related atomic migration mechanism.

Recently, two-dimensional crystalline materials have gained great significance in the design and study of structural and functional materials with novel properties that are absent in three-dimensional materials^8^. In particular, the self-assembly and organization of two-dimensional crystals from micro- and nanosized particles is being used to fabricate tailored materials with specific functionalities^9^,^10^,^11^,^12^. In multicomponent solids, various internal interfaces (e.g., grain boundaries, stacking faults, and twin boundaries) can serve as two-dimensional platforms for the segregation and self-assembly of solute clusters owing to the interaction, disposition, and ordering of the dissolved solute atoms. As a result, the structures of the solute-enriched interfaces go through two-dimensional transitions between thermodynamic states such that the interfacial energy is lowered. Such a nanometer-scale modulated template—atoms and/or nanoparticles of different elements confined in a two-dimensional geometry—promises a great diversity of anisotropic superlattices and phase behaviors. If the phase-like behaviors of interfaces with vastly different properties were to be clarified with respect to the chemical and heat-treatment conditions, it would allow for the control of material properties on a level not previously realizable^13^. A long-range goal in materials science is to develop quantitative and predictive “interface diagrams” as a new tool that aids the fabrication of materials by design^14^.

In this paper, we propose and demonstrate a coarse-grained (CG) modeling approach based on *ab initio* calculations for predicting the equilibrium superlattice structures of solute nanoclusters confined in an atomically close-packed stacking-fault (SF)-type interface. The approach exploits the intriguing solute-enriched layers observed in Mg–M–Y (M = Al or Zn) nanolamellar phases with a long-period stacking order (LPSO)^15^,^16^ as examples of *multicomponent SF complexions*. We investigate the energetic stability and two-dimensional ordering with varying packing density of L1_2_-type core-shell-like M_6_Y_8_ clusters (with diameters of 0.7–0.8 nm) in the SF, depending on the temperature and composition, by considering effective intercluster interactions derived from first-principles calculations based on the density functional theory (DFT). Further, we report nanometer-scale direct observations of solute nanoclusters of a Mg–Zn–Y LPSO phase using scanning tunneling microscopy (STM) to confirm the self-assembly and two-dimensional morphology of Zn–Y clusters along the SF interface in real space. Using the results of quantitative analyses of the STM images in combination with the *ab-initio* CG Monte Carlo (CGMC) calculations, we characterize the positional and orientational orders of the solute clusters in two dimensions, in particular the transformation of the possible local ordering patterns of clusters, considering them analogous to two-dimensional colloidal hard-sphere systems^17^,^18^,^19^.

## SF interfaces in Mg-based nanolamellar LPSO phases

Understanding the behavior of multicomponent SF complexions is of importance for designing metal-based nanolamellar phases, especially those of the (h*_m_*c*_n_*)*_k_* type (here, h and c represent hexagonal and cubic close-packed motifs, respectively; *m*, *n*, and *k* are integers), which are emerging as a new category of nanostructured materials with unique structural and mechanical properties (see, for example, Refs. 20,21,22). In particular, it is worth emphasizing that Mg-based nanolamellar phases with a LPSO structure (hereafter simply called “LPSO phases”) formed in ternary Mg–M–RE (rare earth) alloys have recently attracted significant attention owing to their use in the strengthening of Mg alloys and as promising components for designing advanced lightweight structural materials^15^,^16^,^23^. In such phases, the two-dimensional SF-type interfaces containing solute atoms can play a significant role in the formation of a nanostructured state with low-energy boundaries. As a result, the phases are allowed to contain a high density of SFs and exhibit continuously regular nanolamellar structures formed by SF boundaries, which are an inherent part of their crystal lattice.

**Figure 1:**
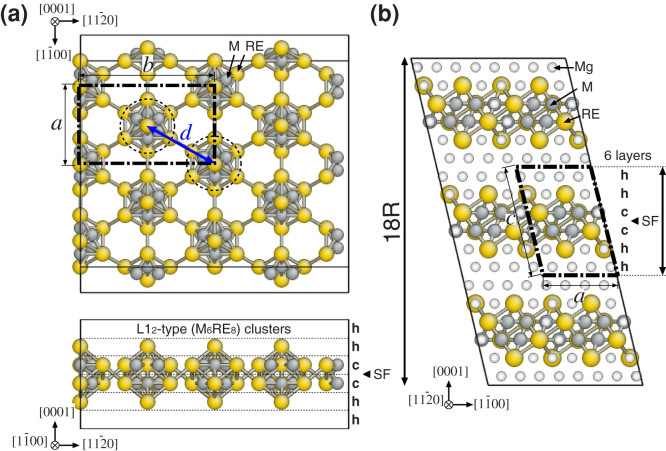
Schematic illustrations of the (a) in-plane and (b) out-of-plane arrangements of M_6_RE_8_ clusters in the 18R-type Mg–M–RE LPSO structure with a complete solute ordering (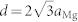). The dashed parallelogram denotes the monoclinic unit cell of the 1M polytype model^24^ with dimensions of *a*, *b*, and *c*. The bars between the solute atoms represent the first nearest-neighbor M–RE and the second nearest-neighbor RE–RE pairs in the Mg matrix.

Recent advances in the analytical techniques such as diffraction, scattering, spectroscopy, microscopy, and imaging have improved our fundamental understanding of the characteristics of ordered arrangements of solute atoms at the internal SF interfaces in ternary Mg–M–RE LPSO alloys^24^,^25^,^26^,^27^. Yokobayashi *et al.*^24^ found that an annealed Mg–Al–Gd ternary alloy exhibited a characteristic solute ordering in the 18R-type (i.e., (hhc|chh)_3_ rhombohedral stacking, where the vertical bar indicates the position of the SF boundary) LPSO phases. In these phases, there exists a six-fold in-plane ordering of the local L1_2_-type Al_6_Gd_8_ clusters at an intercluster distance of 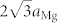 (≈1.1 nm) in the hcch-type close-packed atomic layers containing *I*_2_-type SF (as shown in Fig. 1), where *a*_Mg_ is the unit-cell length along the *a* axis of hexagonal close-packed (hcp) Mg (≈0.32 nm). It was suggested that a local L1_2_-type short-range order, expressed in terms of similar Zn_6_Y_8_ clusters, is also formed in the Mg–Zn–Y system^24^,^25^. Indeed, it was recently reported that a highly ordered Mg–Zn–Y LPSO structure of the 10H-type (i.e., (hhc|ch)_2_ hexagonal stacking) is formed in well-annealed Mg_75_Zn_10_Y_15_ (at.%) alloys, with an analogous six-fold alignment of the Zn_6_Y_8_ clusters noticed along the SF in a periodic manner^27^. However, many of the Mg–Zn–Y LPSO alloys with a lower Zn/Y concentration found so far do not necessarily exhibit such a clear in-plane ordering in electron micrographs^24^,^25^, and their detailed structures at equilibrium remain to be identified. Not only for scientific interests but also for materials design, the inherent natures of heterogeneities and medium-range orders in two-dimensional solute-cluster packing should be elucidated, as this will allow the effective synthesis of versatile Mg–M–RE LPSO structures. This is because solute nanoclusters concurrently having good “stability” and “dispersibility” along the SF will effectively decrease the SF energy with small amounts of M/RE elements and robustly stabilize the nanolamellar structure with a high density of SFs. This, in turn, will affect the overall mechanical properties, thermostability, and chemical resistance of the materials.

## Results

### Coarse-grained Monte Carlo modeling

The strong binding of M/RE atoms to SFs and of M–RE and RE–RE pairs is the major driving force for the formation of the M_6_RE_8_-type nanoclusters in the SF interface in Mg–M–RE LPSO structures^28^,^29^. The binding of solute atoms to SFs, which arises from the difference between the dissolution energies of the solute atom in the hcp layer and in the faulted layer, contributes to the segregation of the solute atoms from the surrounding matrix to the SF interfaces. Further, the attractive interaction of the first nearest-neighbor M–RE and the second nearest-neighbor RE–RE pairs in the close-packed SF interface enhances the formation of the nanosized clusters with an L1_2_-type short-range order^28^.

We had previously pointed out that, on the basis of DFT analyses, the in-plane interaction between L1_2_-type solute clusters in Mg–M–RE LPSO structures could be considered “repulsive”, depending on the element present within the clusters^29^. The DFT results indicated that the significant displacements of RE atoms were induced by the off-lattice relaxation of the L1_2_-type M_6_RE_8_ clusters, especially in the Zn–Y cluster systems, and could be attributed to the transition from a cross-linked (attractive) to unlinked (repulsive) nature of the clusters owing to the change in the electronic structure^29^. Here, we evaluated the pair-wise intercluster interaction energies, which effectively include both chemical and strain-induced contributions, for the Al_6_Y_8_ and Zn_6_Y_8_ clusters in the SF interface on the basis of DFT calculations (details in the Methods section); these are listed in Table 1. Table 1 shows clearly that the intercluster interaction was purely repulsive between the Zn_6_Y_8_ clusters over the range of distances studied (from 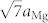 to 4*a*_Mg_) but distinctly attractive between the Al_6_Y_8_ ones at 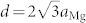, which corresponds to the intercluster separation in the ideal model with a complete solute ordering^24^,^25^,^27^. That the sign of the interaction energy at 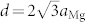 was opposite resulted in an important change in the nature of the in-plane ordering of the clusters, as discussed below. The clear difference in the magnitude between the intracluster energy listed in Supplementary Table S1 online (being of the order of at least −5 eV/cluster as approximated from the formation energies for Mg–M–Y systems containing a cluster) and the intercluster energy listed in Table 1 (at most ±0.1 eV/cluster) warrants a coarse-grained description of the L1_2_-type solute clusters as individual units in the evolution of such systems^28^,^29^. Further, the results of the STM analyses also support the idea of the solute Zn–Y nanoclusters acting as stable and dispersible structural units along the SF interface in the Mg matrix, as described later.

**Figure 2:**
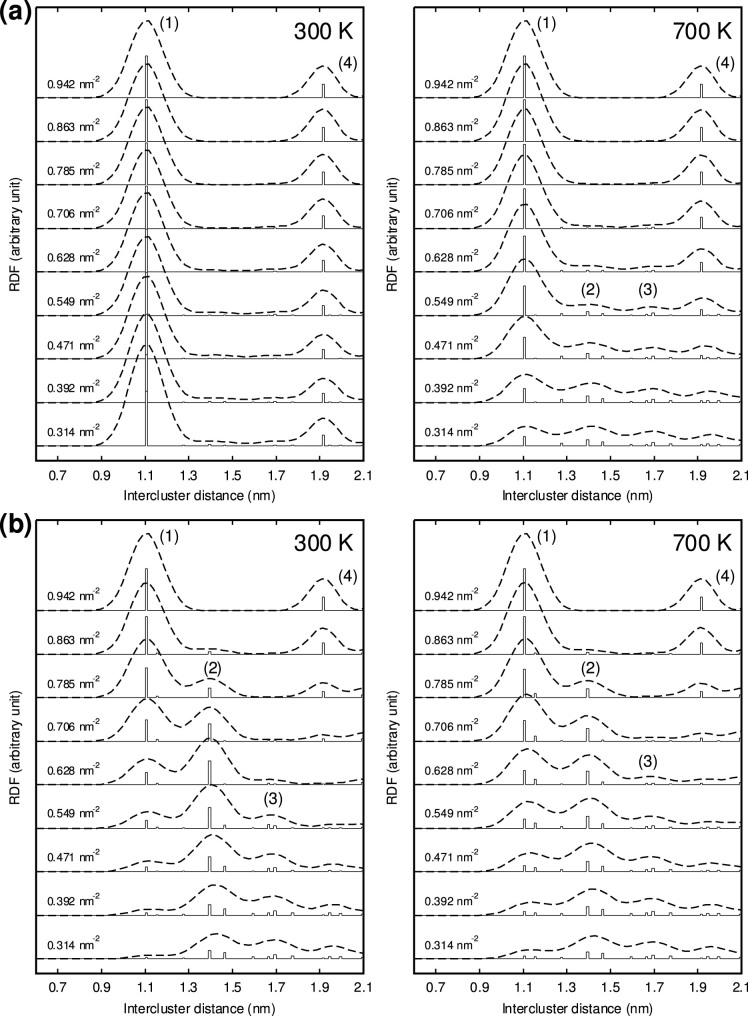
In-plane radial distribution functions (RDFs) of the intercluster distance for (a) Al_6_Y_8_ and (b) Zn_6_Y_8_ cluster systems with various cluster densities, as determined from CGMC simulations. The open bars and dashed lines represent the original RDFs and those smoothened by Bézier curves as visual guides, respectively.

**Figure 3:**
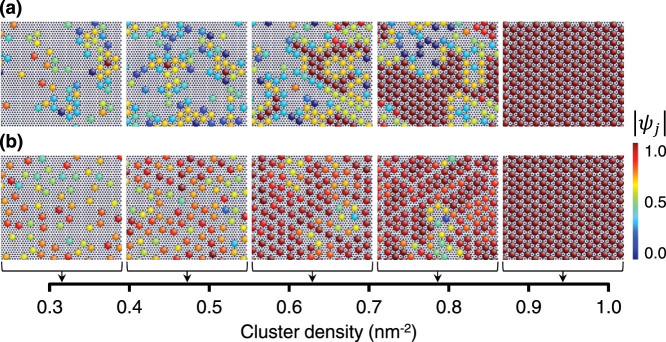
Snapshots of (a) Al_6_Y_8_ and (b) Zn_6_Y_8_ cluster systems obtained through CGMC sampling at 300 K. The systems with a size of 2304 lattice sites (*A* = 15.3 × 13.3 nm^2^) containing 64, 96, 128, 160, and 192 clusters correspond to the cluster densities of 0.314, 0.471, 0.628, 0.785, and 0.942 nm^−2^, respectively. The spheres representing clusters are color-coded with the |*ψ_j_*| value to indicate their degree of six-fold symmetry, which is determined by their neighbors.

**Figure 4:**
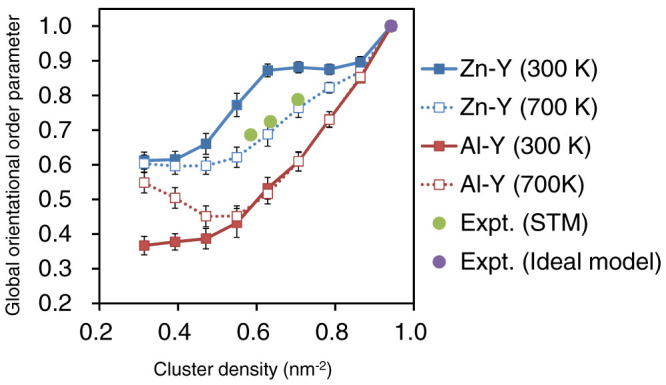
Ensemble-averaged global orientational order parameter of the Al_6_Y_8_ and Zn_6_Y_8_ cluster systems (squares) as a function of the cluster density. The experimental values obtained from the individual STM images (18R1, 18R2, and 18R3) and an ideal model (from Ref. 27) for the Mg–Zn–Y LPSO phase are also plotted for comparison (solid circles).

The in-plane radial distribution functions (RDFs) for the cluster pairs (Fig. 2) and the six-fold orientational order parameters (OOPs) for the individual clusters (Fig. 3) were computed through CGMC simulations (details in the Methods section). We also evaluated the ensemble-averaged values of the global OOP |Ψ_6_| at various cluster densities, as shown in Fig. 4. In Figs. 2 and 3, we observe that the Al–Y and Zn–Y cluster systems both continuously evolve into a densely packed state as the cluster density (*ρ*_clst_) increases. In particular, all the arrangements at *ρ*_clst_ = 0.942 nm^−2^ were found to be the results of the close packing of the clusters at 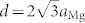 in a given area; this corresponded exactly to the ideal model with a complete in-plane ordering^24^,^25^,^27^, as shown in Fig. 1. The peaks in the RDFs (Fig. 2), which indicate a medium-range positional order beyond the adjacent clusters, become broader and less intense as the cluster density decreases and the temperature increases. For both systems, the first two peaks, at approximately 1.1 and 1.4 nm (labeled 1 and 2, respectively), are characteristic of the local coordination and correspond to the sixth (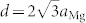) and ninth (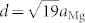) nearest-neighbor positions, respectively, on the triangular lattice with a spacing of *a*_Mg_.

In the Al–Y cluster system, peak 1 is completely dominant and little affected by the change in the cluster density, whereas peak 2 is observed only at 700 K and low *ρ*_clst_ along with peak 1. This indicates that the Al_6_Y_8_ clusters tend to gather near each other even at low cluster densities, owing to the binding between the clusters at 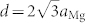, which corresponds to the preferred intercluster distance over others. Thus, the morphology of the clusters is rather heterogeneous because of the coexistence of densely packed domains and regions almost free of clusters, as can be seen in Fig. 3. Further, we found that the global six-fold OOP for the Al–Y cluster system is equal to one only at *ρ*_clst_ = 0.942 nm^−2^ and decreases dramatically as the cluster density decreases, in contrast to that for the Zn–Y cluster system (Fig. 4). This suggests that, in the average structure of the in-plane order in such “attractive” clusters, it is difficult to maintain the overall six-fold symmetry at low-to-medium cluster densities.

Conversely, in the case of the Zn–Y cluster system, a clear trade-off relationship exists between the relative heights of the two peaks in the RDFs; peak 2 steadily emerges at low *ρ*_clst_ and becomes prominent with an increase in the density from *ρ*_clst_ = 0.314 to 0.628 nm^−2^; on the other hand, peak 1 grows and becomes dominant while peak 2 fades away gradually as the density increases further. In Figs. 3 and 4, it is interesting to see that the Zn_6_Y_8_ clusters are positionally less ordered and rather homogeneously dispersed at low *ρ*_clst_, owing to their repulsive interactions; instead, they maintain a high degree of six-fold orientational order (a so-called “hexatic” order^17^,^18^,^19^) over a wide density range. These indicate that the Zn_6_Y_8_ clusters are arranged in mainly two kinds of six-fold domain structures with intercluster distances of 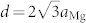 (≈1.1 nm) and 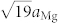 (≈1.4 nm). The transformation from a loosely packed to a densely packed cluster morphology could occur as the cluster density increases while maintaining a high degree of hexatic order. This fact is also confirmed by the behavior of the |Ψ_6_| value for the Zn–Y clusters equilibrated at 300 K, which shows a clear two-step increase with an increase in the cluster density and remains constant at approximately 0.87–0.90 in the intermediate range of *ρ*_clst_ from 0.628 to 0.863 nm^−2^, as shown in Fig. 4.

### Scanning tunneling microscopy

STM has the ability to map spatially inhomogeneous electronic systems as well as image spatial modulations on the surfaces of the heterogeneous atomic structures in the systems. We had previously reported that two-dimensional images of the cluster arrangements at the SF interface in the Mg–Zn–Y LPSO phase could be obtained using STM^30^. In these images, high-contrast spots with a diameter of <1 nm, each of which was assumed to be a Zn–Y cluster, were hexagonally arranged along the (0001)_hcp_ plane and organized into domains, whose sizes were estimated to typically range from several nanometers to a few tens of nanometers. In addition, the structures at the domain boundaries of the clusters on the triangular lattice were imaged and identified using STM^30^.

During this analysis, we investigated the heterogeneous features of the chemically modulated nanostructures, especially the spatially ordered patterns of the nanoclusters dispersed parallel to the SF interface, on the (0001)_hcp_ cleavage surface of the 18R-type LPSO phases in a Mg_85_Zn_6_Y_9_ (at.%) alloy using STM. Three fields of view were chosen for the sample, and the two-dimensional positions of the clusters were determined from the STM images by identifying the centers of the dark contrast spots in these areas via digital image processing; cluster arrangements comprising 478, 394, and 251 clusters were examined, and their densities were estimated to be (a) 0.69–0.72 nm^−2^, (b) 0.62–0.65 nm^−2^, and (c) 0.57–0.60 nm^−2^ (hereafter labeled as “18R1”, “18R2”, and “18R3”), respectively. The distortions in the images due to thermal drift were removed by assuming the ideal model of the LPSO structure. The noise was also removed, using low-pass filtering.

**Figure 5:**
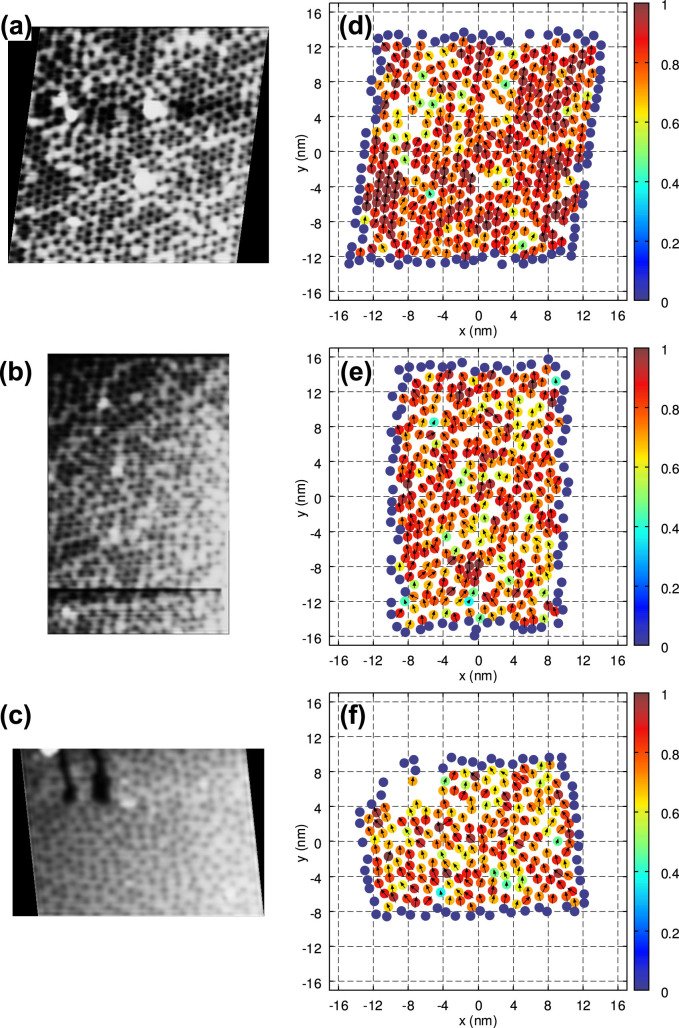
STM results of the cluster arrangements in the 18R-type LPSO phase inside a Mg_85_Zn_6_Y_9_ (at.%) alloy. (a–c) STM images of the cleaved surface of the LPSO phase obtained by applying a positive bias voltage on the sample. Three kinds of cluster arrangements were noticed in the STM images (18R1, 18R2, and 18R3), and their cluster densities were measured to be (a) 0.69–0.72 nm^−2^, (b) 0.62–0.65 nm^−2^, and (c) 0.57–0.60 nm^−2^, respectively. The tunneling parameters are: (a) *V_s_* = 0.7 V, *I_t_* = 1.5 nA; (b, c) *V_s_* = 0.8 V, *I_t_* = 1.0 nA. (d–f) cluster arrangements extracted from the STM images (color-coded by the |*ψ_j_*| value) and their local orientational order parameter fields represented by the vectors (Re(*ψ_j_*), Im(*ψ_j_*)). The clusters in the edge region (dark-blue circles) are treated as boundaries and excluded from the calculation of the averages.

**Figure 6:**
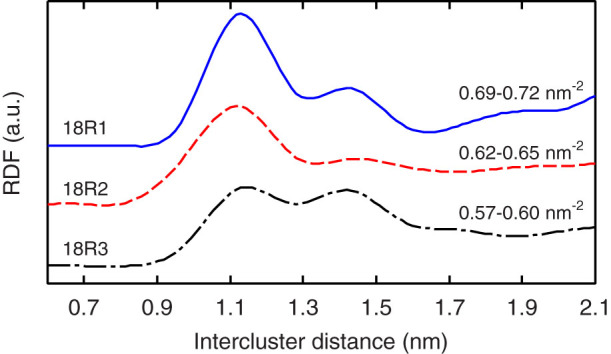
In-plane radial distribution functions of the intercluster distance for the 18R1, 18R2, and 18R3 arrangements at *ρ*_clst_ = 0.69–0.72 nm^−2^, 0.62–0.65 nm^−2^, and 0.57–0.60 nm^−2^, respectively, obtained from the STM images.

Figure 5 shows the STM images of the 18R1, 18R2, and 18R3 arrangements and the extracted positions of their solute clusters. Further, the six-fold OOPs and the in-plane RDFs were also examined (Figs. 5 and 6) to obtain information regarding the positional and orientational orders of the solute clusters in each arrangement. In Fig. 5, we can observe the lattice-like nanostructures with a locally heterogeneous distribution of the clusters, which are mostly arranged in a hexagonal manner but are not regularly and periodically ordered. In particular, while a large number of densely packed clusters were found at a high packing fraction (18R1), the clusters seemed to widen the space between the neighbors at a lower density (18R2 and 18R3). The RDFs in Fig. 6 indicate that the two characteristic peaks, which represent the preferred intercluster distances, were observed at approximately 1.1 nm and 1.4 nm, respectively. Note that the intercluster distance of 1.1 nm corresponds to that in the ideal model with a complete ordering of the L1_2_-type solute clusters^24^,^25^,^27^. With an increase in the cluster density (i.e., 18R3 → 18R2 → 18R1), the height of the first peak (≈1.1 nm) increases relative to that of the second peak (≈1.4 nm), while the position of the first peak is gradually shifted to the left. Such a behavior suggests that the organization of the cluster morphology in the system may not be caused by strong binding at a certain distance between the clusters, but by a change in the packing geometry in a rather “push-and-shove” manner as the cluster density increases. As a result, the cluster morphology consists of multiple (i.e., at least two) kinds of six-fold ordering units with different intercluster distances in two dimensions, each of which contributes to the two broad peaks at approximately 1.1 and 1.4 nm in the RDFs, and their population ratio can change with an increase/decrease in the cluster density.

Further, the local coordination structures of the neighboring clusters were characterized using a six-fold OOP *ψ_j_* (Fig. 5). We found that each cluster maintained a high |*ψ_j_*| value and that the OOP vectors (Re(*ψ_j_*), Im(*ψ_j_*)) were loosely aligned with those of the neighbors in nearly the same direction; consequently, the OOPs appear to be correlated in almost all the fields comprising several hundreds of clusters. It is interesting to note that the orientational orders in the cluster arrangements are quasi-long range whereas their positional orders remain short range, as in the intermediate hexatic phase of colloidal hard-sphere systems^17^,^18^,^19^.

## Discussion

It is notable that the employed CG model, which was based on DFT calculations, for the Zn–Y cluster system provided results that were in good agreement with the results of the STM analyses. This implies that the intercluster interaction evaluated here could correctly extract the essential features of the in-plane ordering of the solute nanoclusters with a “hard-sphere-like” character in the Mg matrix. In particular, the CGMC simulations at 700 K were able to reproduce not only the overall variation in the relative heights of the two characteristic peaks in the RDFs (Fig. 6) but also the global six-fold OOPs (Fig. 4) seen in the STM results at the experimental cluster densities. This fact suggests the possibility that, owing to the rapid quenching of the specimen in air from the solidification temperature, the cluster morphology formed at a high temperature was kinetically frozen and its original shape was retained throughout the experiment. Our findings indicate that the increase in the volume fraction of the solute clusters results in a continuous transformation with an increase in the hexatic order in a push-and-shove manner, which can explain the steady reduction of the radial correlation length between the clusters with maintaining a six-fold-like pattern that was observed in synchrotron radiation small-angle X-ray scattering measurements for Mg_85_Zn_6_Y_9_ (at.%) LPSO alloys during the annealing at high temperatures^26^.

Note that such predicted ordering patterns of the Zn–Y clusters in Figs. 2(b) and 3(b) have a mainly entropic origin; there are no significant attractive interactions among the clusters. Generally, hexatic ordering occurs in a two-dimensional hard-sphere system because the loss of the configurational (orientational) entropy of the particles in a dense packing is overcompensated by the high translational (positional) entropy of the particles^17^. In this sense, the ordering behavior of the attractive Al–Y clusters is dominated by the chemical trapping of clusters by the neighboring bonds, whereas that of the repulsive Zn–Y clusters is dominated by the topological trapping of clusters by each other in cages. Such an ordering mechanism of the cluster arrangements resulting from the entropic effect can be expected to be robust and flexible enough to allow fluctuations in the composition and stoichiometry for the multicomponent interfaces formed in Mg–M–RE nanolamellar phases.

In this study, the experimental analysis on the spatially ordered patterns of the clusters was based on the premise that the dark contrast spots observed in the STM images corresponded to the Zn–Y nanoclusters of the L1_2_-type and its derivatives. It was most probable that an accumulation of electrons around the clusters, which was caused by the strong chemical bonds formed inside the cluster by the Zn/Y atoms with relatively large atomic number, contributed to the origin of the contrast in the STM images. Also, the size, density, and geometric patterns of dark contrast spots were consistent with those in the solute-cluster superlattices observed in other experimental studies^24^,^25^,^26^,^27^,^31^. Nevertheless, the careful investigation of the electronic structure, in particular the local density of states close to the Fermi level, is preferably required to clarify the contrast mechanism and to identify chemical species in the STM images of the specimen. Such an analysis will be the subject of the future work.

In summary, the computational approach employed in this study could successfully predict the nature of two-dimensionally ordered arrangements of Zn–Y nanoclusters in a Mg-based nanolamellar phase, in a manner consistent with STM observations performed in real space. This combined computational-experimental analysis provides clear insights into the phase-like behavior of a close-packed SF interface showing a disorder-order transition in a solute-cluster superlattice. In addition, the controversial issue of the composition dependence of in-plane orderings in the Mg–M–Y LPSO phases was resolved by examining the characteristics of the ordering behavior of attractively or repulsively interacting nanoclusters in the SF interface. We predicted that, as the number of clusters per SF interface is increased (owing to solute segregation and clustering at high temperatures), the system undergoes a continuous evolution into a highly ordered densely packed one while maintaining a high degree of six-fold orientational order, which is attributable mainly to an entropic effect. This approach should help engineer nanometer-scale chemical modulation and ordering in metal-based nanolamellar systems^20^,^21^,^22^.

## Methods

### *Ab initio* intercluster interaction

The energetics and equilibrium structure of the Mg-based LPSO phases containing L1_2_-type M_6_Y_8_ (M = Al or Zn) clusters were obtained from DFT calculations with the projector-augmented-wave method^32^,^33^ using the Vienna Ab initio Simulation Package (VASP)^34^. We applied the generalized gradient approximation of the Perdew–Wang^35^ form for the exchange-correlation functional in DFT. The 18R-type LPSO structures with various intercluster distances (*d*) were modeled using 84- to 192-atom triclinic (or monoclinic) cells with lattice constants of *a* = *d* and 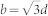 (or vice versa) containing an *I*_2_-type intrinsic SF and M_6_Y_8_ cluster(s) at the middle of their unit cells (see Supplementary Fig. S1 online), which are formed with only a single six-layer structural block—i.e., through hhcchh stacking—by choosing its stacking vector to be the *c* axis of the triclinic (or monoclinic) cell. Note that the 144-atom cell (i.e., Mg_116_M_12_Y_16_ crystal) corresponds to an ideal model with a complete solute ordering at 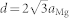 that stems from the 1M polytype (space group of *C*2/*m*)^24^,^25^ for the 18R-type Mg–M–RE order-disorder structures. Calculations to determine the formation energies and the intercluster interaction were performed using these unit cells of the Mg–M–Y system, with a plane-wave energy cutoff of 345.9 eV using a Monkhorst–Pack^36^ *k*-point mesh for integration over the Brillouin zone and a Methfessel–Paxton smearing method^37^ with a width of 0.2 eV, while using the tetrahedron method with a Blöchl correction^38^ to determine the total energies. Structural relaxations were performed until the forces on each atom were less than 3 meV/Å.

The formation energies (*E*_f_) of the Mg–M–Y LPSO structures are defined as follows: 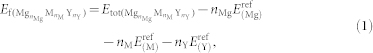
where the *E*_tot_ represents the DFT-calculated total energies of the cells, *n*_Mg_, *n*_M_, and *n*_Y_ refer to the numbers of Mg, M, and Y atoms in each unit cell, and *N* is the total number of atoms in the cell so that *N* = *n*_Mg_ + *n*_M_ + *n*_Y_. Note that *E*_f_ is with respect to the reference energy *E*^ref^ of pure Mg, M, and Y in its equilibrium structure (hcp for Mg, Zn, and Y and face-centered cubic for Al). Supplementary Table S1 lists the *E*_f_ values and lattice parameters of 18R-type Mg–M–Y LPSO structures with various intercluster distances obtained from the DFT calculations.

We defined the pair-interaction energies between the M_6_Y_8_ clusters of L1_2_ type as follows: 
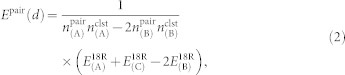
where *d* is the intercluster distance ranging from 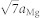 to 4*a*_Mg_ in the *I*_2_ SF layer; *E*^18R^ represents the total energy for the 18R-type layer structure, with the letters A, B, and C representing the composition of the unit cell containing the same number of atoms *N*, i.e., A = Mg*_N_*_−28_M_12_Y_16_, B = Mg*_N_*_−14_M_6_Y_8_, and C = Mg*_N_*, respectively. The systems with compositions A and B contain two and one M_6_Y_8_ cluster(s) with six-fold and two-fold coordination, respectively (Supplementary Fig. S1), in the hcch-type stacking layers at the middle of their unit cells. Since all the 18R-type layer structures studied were modeled using a single six-layer structural block with dimensions of 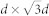 (as in Fig. 1), *N* is defined as a function of *d* such that *N* = 12(*d*/*a*_Mg_)^2^. *n*^pair^ is the number of intercluster pairs (per cluster) in the unit cell and *n*^clst^ is the number of clusters in the unit cell, so that 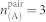, 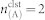, 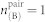, and 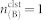. Here, we suppose that the contribution of each intercluster pair (at the same *d*) to the interaction energy is equivalent to that of the others. The bracket in equation (2) represents a part of the interaction energy between clusters, which is extracted in a pair-wise fashion from the cluster arrangements with different coordination numbers (see Supplementary Fig. S2 online). This value is divided by the net number of intercluster pairs to obtain the contribution of a single pair interaction (*E*^pair^). Table 1 lists the *E*^pair^ values obtained from the DFT calculations. Note that a negative value of *E*^pair^ corresponds to an attractive interaction under this definition.

### Coarse-grained Monte Carlo modeling

In this study, the M_6_Y_8_ clusters were modeled as single CG particles with a chosen core radius to prevent them from coalescing with the other clusters. The CG particles interact with each other through the pair potential, which consists of a short-range soft-core repulsion—modeled as an artificial energy barrier up to a few eV within a core radius of 2*a*_Mg_, corresponding to the third nearest-neighbor distance in the triangular lattice with a spacing of *a*_Mg_—and a longer-range intercluster energy evaluated from the DFT calculations, as listed in Table 1. Consequently, the CG particles can be prevented from overlapping and coalescing with each other, while being kept at an excluded distance of 2*a*_Mg_ in the configurations equilibrated at the target temperatures.

For the equilibrium simulations of the CG cluster model, 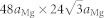 (i.e., 15.34 nm × 13.29 nm) two-dimensional triangular lattices containing 2304 lattice sites were used. These correspond to the 13824-atom system of the 18R-type LPSO structure (here assumed to comprise a single six-layer structural block) based on the all-atom representation. As an initial configuration, CG particles expressing M_6_Y_8_ clusters were randomly distributed on the sites of a triangular lattice with a spacing of *a*_Mg_ with a composition of Mg–2.8at.%M–3.7at.%Y (i.e., 64 clusters) to Mg–8.3at.%M–11.1at.%Y (i.e., 192 clusters) with an M/Y ratio of 3/4, where the cluster density ranges from 0.314 to 0.942 nm^−2^. The remaining sites were alternatively occupied by “virtual” particles that represent vacant lattice points. Thermal equilibrations of the M–Y cluster systems were carried out using Markov chain Monte Carlo simulations without detailed balance^39^,^40^ in the canonical ensemble, via successive exchanges of pairs of neighboring particles under periodic boundary conditions. The simulations, which started with multiple (typically 10) initial configurations with different sets of random positions of solute clusters, were performed for 4 × 10^6^ steps at a sufficiently high temperature (e.g., 2700 K) to homogenize the distribution of the clusters. After that, the system was equilibrated for 4 × 10^6^ steps at 700 K, and then further cooled to 300 K for 4 × 10^6^ steps.

### Positional order parameter

The positional order of the cluster system can be characterized by an in-plane radial distribution function *g*(*r*) for clusters. This function tells us where the clusters are relative to each other along the radius, *r*. The function *g*(*r*) is defined as the distribution of cluster pairs at a distance *r* = |***x****_i_* − ***x****_j_*|, normalized such that *g*(*r* → ∞) = 1. In practice, the intercluster distances are binned into a histogram with a bin size Δ*r*. If *n* out of the *p* distance pairs are found to lie in the interval [*r* − Δ*r*/2, *r* + Δ*r*/2], then we have 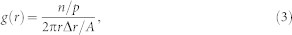
where *A* is the area of an SF interface along which clusters are dispersed. The ensemble-averaged *g*(*r*) for the clusters is calculated by taking an average over the configurations generated from multiple CGMC runs at a constant temperature.

### Orientational order parameter

As in a two-dimensional hard-sphere system, the global orientational order parameter^18^,^19^ of the CG cluster system is given by 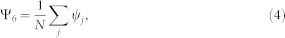
which is the spatial average of the local orientational order parameter 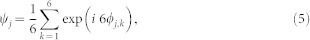
where 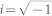. The sum is over the six closest neighbors *k* of the cluster *j*, and *φ_j,k_* is the angle between the shortest periodic vector equivalent to ***x****_k_* − ***x****_j_* and an arbitrary but fixed reference vector. The value of |*ψ_j_*| = |Ψ_6_| is equal to one if all the clusters sit on a perfect triangular lattice, whereas it becomes nearly zero when the structure is disordered. *ψ_j_* can be represented as a vector (Re(*ψ_j_*), Im(*ψ_j_*)) and rotates by 2*π* if the lattice is rotated by an angle of *π*/3 without changing the reference vector.

### Scanning tunneling microscopy

A master ingot of Mg_85_Zn_6_Y_9_ (at.%) was prepared by induction melting in a carbon crucible and then directionally solidified in a furnace (NEV-DS2, Nissin Giken) using the Bridgman technique at a solidification rate of 10 mm h^−1^ in an Ar atmosphere. It was confirmed that the volume fraction of the 18R-type LPSO phases was almost 100%^23^. To evaluate a specimen through STM, we prepared atomically flat surfaces using the cleavage method^41^,^42^. The specimen was fabricated from a slice of the alloy ingot with a thickness of 0.5 mm. A notch was made, and the specimen was cleaved at the liquid-nitrogen temperature. When an external lateral force was applied to the notched specimen at this temperature, the specimen fractured into two pieces in a brittle manner. Most of the fracture surface was not flat on the nanometer scale owing to its polycrystalline nature; however, a part of the surface exhibited an atomically smooth terraces-and-steps structure^30^. The STM observations were performed with a USM-1200 (Unisoku) at the liquid-nitrogen temperature. These procedures were performed in a ultrahigh-vacuum chamber (base pressure ≈ 1.0 × 10^−8^ Pa) to prevent the oxidation of the surface.

We assumed that the structure of the imaged surface of the specimen originated from the sequential cleaving of the preferred crystallographic planes, most likely along the close-packed (0001)_hcp_ planes with the lowest surface energy in the hcp Mg layers (confined between the two SF interfaces), where the enrichment of clusters does not occur. This is principally because the non-basal slips, twins, and kinks are unlikely to be activated at an extremely low temperature (≈77 K), owing to the high formation energy of the solute clusters and the severe limitation of the deformation modes in the Mg–Zn–Y LPSO phases^23^.

The specimen was rapidly cooled from the melting temperature (about 893 K^31^) to ambient temperature no longer than a couple of hours during the solidification process. In addition, for comparison, we prepared a different specimen cut from a non-directionally solidified (i.e., as-cast) Mg_85_Zn_6_Y_9_ (at.%) alloy ingot, and then confirmed that there was no significant difference in the characteristics of the in-plane order in cluster arrangements at a similar cluster density.

## Author Contributions

H.K. and S.K. designed the study and drafted the manuscript. H.K. carried out the numerical simulations and analyzed the data. S.K. and A.Y. carried out the experiments and analyzed the data. A.S. and S.O. supervised the work and provided critical feedback on the manuscript. All authors contributed to discussion of the results.

## Supplementary Material

Supplementary Information Supplementary Information for Two-Dimensional Ordering of Solute Nanoclusters at a Close-Packed Stacking Fault: Modeling and Experimental Analysis

## Figures and Tables

**Table 1:** Pair-wise intercluster interaction energy for the Al_6_Y_8_ and Zn_6_Y_8_ clusters in the stacking-fault interface. A positive energy corresponds to a repulsive interaction. The intercluster distance *d* is defined as the distance between the clusters that occupy the *l*-th nearest neighbor (NN) sites on the triangular lattice with a spacing of *a*_Mg_

Configuration				*E*^pair^ [eV]	Intercluster
Site (NN)	*d* [*a*_Mg_]		Al_6_Y_8_	Zn_6_Y_8_	geometry
0	0		∞	∞	Overlapped
1	1		—	—	Coalesced
2	1.73	( 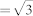 )	—	—	Coalesced
3	2		—	—	Coalesced
4	2.65	( 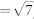 )	0.595	0.426	Contacted
5	3		0.213	0.411	Contacted
6	3.46	( 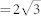 )	−0.029	0.025	Contacted
7	3.61	( 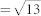 )	0.110	0.063	Dissociated
8	4		−0.004	0.030	Dissociated
