# Supplementary material for: Two-Dimensional Ordering of Solute Nanoclusters at a Close-Packed Stacking Fault: Modeling and Experimental Analysis
Source: Sci Rep. 2014 Dec 4;4:7318. doi: 10.1038/srep07318 (PMC4255191; doi:10.1038/srep07318)
Supplement: Supplementary Information — for Two-Dimensional Ordering of Solute Nanoclusters at a Close-Packed Stacking Fault: Modeling and Experimental Analysis [file srep07318-s1.pdf]

Supplementary Information

Two-Dimensional Ordering of Solute Nanoclusters at a Close-Packed Stacking Fault: Modeling and Experimental Analysis

Hajime Kimizuka,[1,*] Shu Kurokawa,[2,3,†]

Akihiro Yamaguchi,[2] Akira Sakai,[2] and Shigenobu Ogata[1,3]

[1]*Department of Mechanical Science and Bioengineering, Osaka University, Osaka 560-8531, Japan*

[2]*Department of Materials Science and Engineering, Kyoto University, Kyoto 606-8501, Japan*

[3]*Center for Elements Strategy Initiative for Structural Materials, Kyoto University, Kyoto 606-8501, Japan*

* kimizuka@me.es.osaka-u.ac.jp (Corresponding author)

† kurokawa.shu.4m@kyoto-u.ac.jp (Corresponding author)

Supplementary Table S1. Formation energies and lattice parameters of 18R-type Mg–M–Y LPSO structures with various intercluster distances. Compositions A and B denote the systems of $Mg_{N-28}M_{12}Y_{16}$ ($n^{\mathrm{pair}} = 3$, $n^{\mathrm{clst}} = 2$) and $Mg_{N-14}M_6Y_8$ ($n^{\mathrm{pair}} = 1$, $n^{\mathrm{clst}} = 1$), respectively. d represents the in-plane intercluster distance.

System	# atoms (N)	d [a_{Mg}]	# k-points	E_{f} [eV]	Lattice parameters (relaxed)		
Composition A (M = Al)							
$Mg_{56}Al_{12}Y_{16}$	84	$\sqrt{7}$	$10 \times 16 \times 9$	−7.71	$a = 1.477$ nm,<br>$\alpha = 81.62°$,	$b = 0.857$ nm,<br>$\beta = 99.62°$,	$c = 1.617$ nm<br>$\gamma = 90.18°$
$Mg_{80}Al_{12}Y_{16}$	108	3	$8 \times 14 \times 9$	−9.95	$a = 1.670$ nm,<br>$\alpha = 90°$,	$b = 0.966$ nm,<br>$\beta = 103.04°$,	$c = 1.614$ nm<br>$\gamma = 90°$
$Mg_{116}Al_{12}Y_{16}$	144	$2\sqrt{3}$	$12 \times 7 \times 9$	−11.22	$a = 1.115$ nm,<br>$\alpha = 90°$,	$b = 1.929$ nm,<br>$\beta = 103.54°$,	$c = 1.604$ nm<br>$\gamma = 90°$
$Mg_{128}Al_{12}Y_{16}$	156	$\sqrt{13}$	$7 \times 12 \times 9$	−10.04	$a = 2.020$ nm,<br>$\alpha = 86.82°$,	$b = 1.166$ nm,<br>$\beta = 102.90°$,	$c = 1.611$ nm<br>$\gamma = 90.00°$
$Mg_{164}Al_{12}Y_{16}$	192	4	$6 \times 10 \times 9$	−10.89	$a = 2.227$ nm,<br>$\alpha = 90°$,	$b = 1.286$ nm,<br>$\beta = 103.47°$,	$c = 1.607$ nm<br>$\gamma = 90°$
Composition B (M = Al)							
$Mg_{70}Al_6Y_8$	84	$\sqrt{7}$	$10 \times 16 \times 9$	−4.93	$a = 1.474$ nm,<br>$\alpha = 81.12°$,	$b = 0.859$ nm,<br>$\beta = 99.83°$,	$c = 1.611$ nm<br>$\gamma = 90.20°$
$Mg_{94}Al_6Y_8$	108	3	$8 \times 14 \times 9$	−5.26	$a = 1.667$ nm,<br>$\alpha = 90°$,	$b = 0.962$ nm,<br>$\beta = 103.53°$,	$c = 1.607$ nm<br>$\gamma = 90°$
$Mg_{130}Al_6Y_8$	144	$2\sqrt{3}$	$12 \times 7 \times 9$	−5.35	$a = 1.110$ nm,<br>$\alpha = 90°$,	$b = 1.922$ nm,<br>$\beta = 103.43°$,	$c = 1.604$ nm<br>$\gamma = 90°$
$Mg_{142}Al_6Y_8$	156	$\sqrt{13}$	$7 \times 12 \times 9$	−5.02	$a = 2.002$ nm,<br>$\alpha = 87.17°$,	$b = 1.155$ nm,<br>$\beta = 102.96°$,	$c = 1.603$ nm<br>$\gamma = 89.85°$
$Mg_{178}Al_6Y_8$	192	4	$6 \times 10 \times 9$	−5.17	$a = 2.218$ nm,<br>$\alpha = 90°$,	$b = 1.281$ nm,<br>$\beta = 103.32°$,	$c = 1.602$ nm<br>$\gamma = 90°$
Composition A (M = Zn)							
$Mg_{56}Zn_{12}Y_{16}$	84	$\sqrt{7}$	$10 \times 16 \times 9$	−8.35	$a = 1.489$ nm,<br>$\alpha = 81.21°$,	$b = 0.861$ nm,<br>$\beta = 100.08°$,	$c = 1.618$ nm<br>$\gamma = 89.94°$
$Mg_{80}Zn_{12}Y_{16}$	108	3	$8 \times 14 \times 9$	−8.78	$a = 1.690$ nm,<br>$\alpha = 90°$,	$b = 0.976$ nm,<br>$\beta = 103.42°$,	$c = 1.608$ nm<br>$\gamma = 90°$
$Mg_{116}Zn_{12}Y_{16}$	144	$2\sqrt{3}$	$12 \times 7 \times 9$	−10.77	$a = 1.116$ nm,<br>$\alpha = 90°$,	$b = 1.936$ nm,<br>$\beta = 103.55°$,	$c = 1.608$ nm<br>$\gamma = 90°$
$Mg_{128}Zn_{12}Y_{16}$	156	$\sqrt{13}$	$7 \times 12 \times 9$	−10.45	$a = 2.013$ nm,<br>$\alpha = 86.78°$,	$b = 1.162$ nm,<br>$\beta = 102.88°$,	$c = 1.606$ nm<br>$\gamma = 90.05°$
$Mg_{164}Zn_{12}Y_{16}$	192	4	$6 \times 10 \times 9$	−10.63	$a = 2.228$ nm,<br>$\alpha = 90°$,	$b = 1.288$ nm,<br>$\beta = 103.50°$,	$c = 1.606$ nm<br>$\gamma = 90°$
Composition B (M = Zn)							
$Mg_{70}Zn_6Y_8$	84	$\sqrt{7}$	$10 \times 16 \times 9$	−4.91	$a = 1.470$ nm,<br>$\alpha = 81.05°$,	$b = 0.855$ nm,<br>$\beta = 99.98°$,	$c = 1.609$ nm<br>$\gamma = 90.07°$
$Mg_{94}Zn_6Y_8$	108	3	$8 \times 14 \times 9$	−5.07	$a = 1.662$ nm,<br>$\alpha = 90°$,	$b = 0.969$ nm,<br>$\beta = 103.57°$,	$c = 1.608$ nm<br>$\gamma = 90°$
$Mg_{130}Zn_6Y_8$	144	$2\sqrt{3}$	$12 \times 7 \times 9$	−5.24	$a = 1.112$ nm,<br>$\alpha = 90°$,	$b = 1.921$ nm,<br>$\beta = 103.51°$,	$c = 1.607$ nm<br>$\gamma = 90°$
$Mg_{142}Zn_6Y_8$	156	$\sqrt{13}$	$7 \times 12 \times 9$	−5.14	$a = 2.001$ nm,<br>$\alpha = 86.96°$,	$b = 1.157$ nm,<br>$\beta = 102.86°$,	$c = 1.604$ nm<br>$\gamma = 89.93°$
$Mg_{178}Zn_6Y_8$	192	4	$6 \times 10 \times 9$	−5.11	$a = 2.216$ nm,<br>$\alpha = 90°$,	$b = 1.284$ nm,<br>$\beta = 103.37°$,	$c = 1.604$ nm<br>$\gamma = 90°$
Composition C							
Mg_{84}	84		$10 \times 16 \times 9$	0.23	$a = 1.464$ nm,<br>$\alpha = 81.32°$,	$b = 0.845$ nm,<br>$\beta = 100.04°$,	$c = 1.600$ nm<br>$\gamma = 90.00°$
Mg_{108}	108		$8 \times 14 \times 9$	0.29	$a = 1.660$ nm,<br>$\alpha = 90°$,	$b = 0.958$ nm,<br>$\beta = 103.32°$,	$c = 1.601$ nm<br>$\gamma = 90°$
Mg_{144}	144		$12 \times 7 \times 9$	0.40	$a = 1.106$ nm,<br>$\alpha = 90°$,	$b = 1.916$ nm,<br>$\beta = 103.33°$,	$c = 1.600$ nm<br>$\gamma = 90°$
Mg_{156}	156		$7 \times 12 \times 9$	0.43	$a = 1.995$ nm,<br>$\alpha = 86.83°$,	$b = 1.151$ nm,<br>$\beta = 102.93°$,	$c = 1.600$ nm<br>$\gamma = 90.00°$
Mg_{192}	192		$6 \times 10 \times 9$	0.53	$a = 2.212$ nm,<br>$\alpha = 90°$,	$b = 1.278$ nm,<br>$\beta = 103.33°$,	$c = 1.601$ nm<br>$\gamma = 90°$

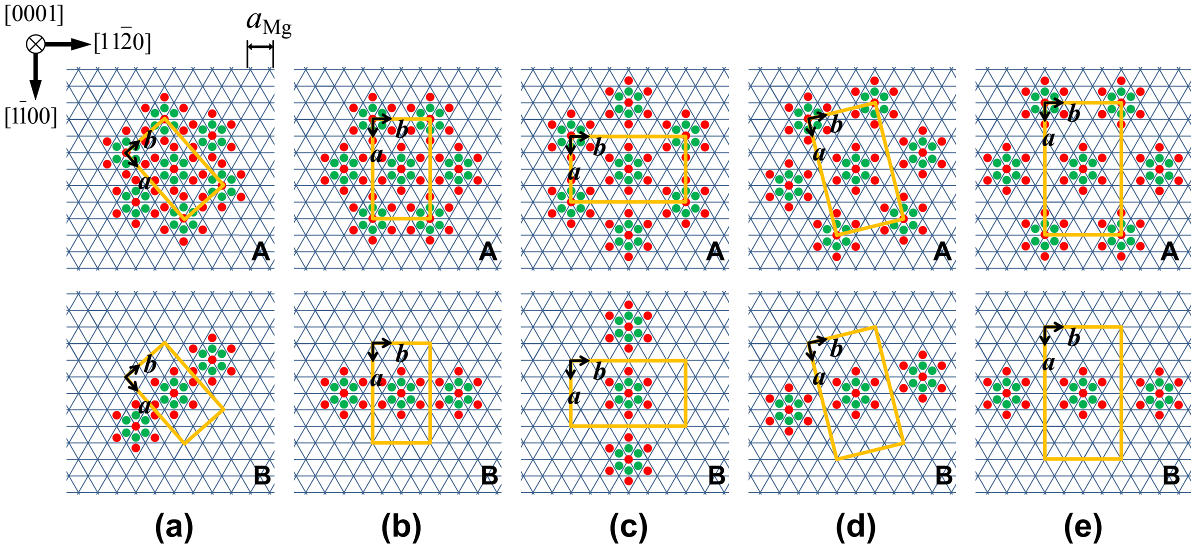

Supplementary Figure S1. Schematic of an in-plane periodic arrangement of M_6Y_8 clusters along the stacking-fault interface in the $Mg_{N-28}M_{12}Y_{16}$ (composition A: upper panels) and $Mg_{N-14}M_6Y_8$ (composition B: lower panels) systems with an intercluster distance of (a) $\sqrt{7}a_{\mathrm{Mg}}$, (b) $3a_{\mathrm{Mg}}$, (c) $2\sqrt{3}a_{\mathrm{Mg}}$, (d) $\sqrt{13}a_{\mathrm{Mg}}$, and (e) $4a_{\mathrm{Mg}}$, respectively. Only the solute atoms are displayed: M atoms in green and Y atoms in red solid circles.

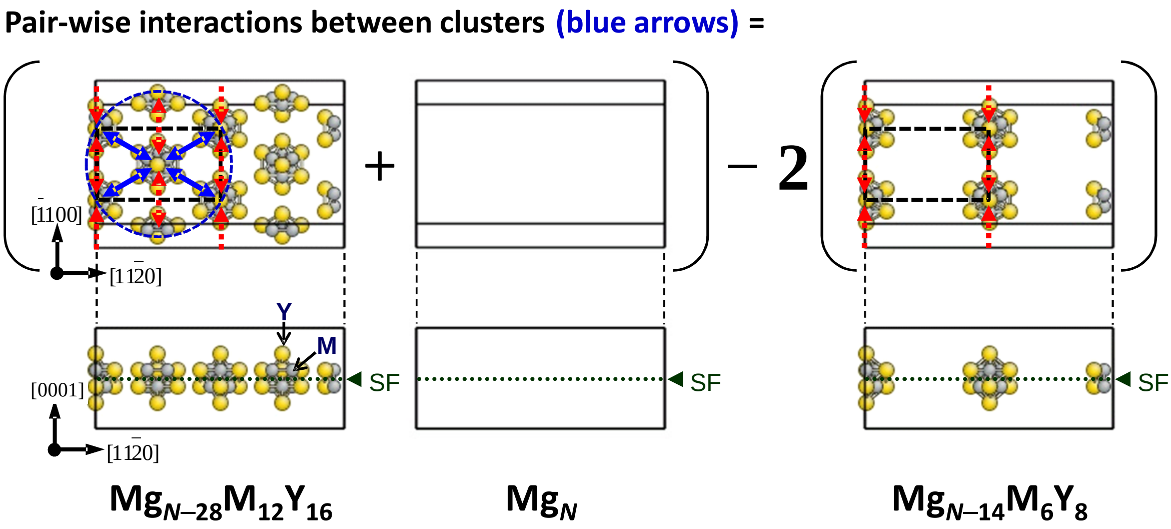

Supplementary Figure S2. Schematic of an in-plane periodic arrangement of M_6Y_8 clusters along the stacking fault layers in the $Mg_{N-28}M_{12}Y_{16}$ (composition A), $Mg_{N-14}M_6Y_8$ (composition B), and Mg_N (composition C) systems. Only the solute atoms are displayed: M atoms in gray and Y atoms in yellow spheres. Blue and red arrows represent pair-wise interactions inside the cluster-coordination shells with a radius of d.
